# The Age-Related Efficacy of Dimethyl Fumarate and Natalizumab in the Real-World Management of Multiple Sclerosis

**DOI:** 10.3390/ph14020081

**Published:** 2021-01-22

**Authors:** Roberto De Masi, Stefania Orlando, Antonella De Donno

**Affiliations:** 1 Laboratory of Neuroproteomics, Multiple Sclerosis Centre, “F. Ferrari” Hospital, 73042 Casarano, Lecce, Italy; dmsrrt@gmail.com; 2 Complex Operative Unit of Neurology, “F. Ferrari” Hospital, 73042 Casarano, Lecce, Italy; 3 Laboratory of Hygiene, Department of Biological and Environmental Sciences and Technologies, University of the Salento, 73100 Lecce, Italy; antonella.dedonno@unisalento.it

**Keywords:** dimethyl fumarate, natalizumab, drug efficacy, multiple sclerosis

## Abstract

We investigated the comparative age-related efficacy of dimethyl fumarate (DMF) and natalizumab (NTZ) in clinical practice on multiple sclerosis (MS). Research in this area is lacking in the previous literature. In a three-year retrospective and clinical–paraclinical study, we compared 173 DMF patients and 94 NTZ patients with a similar average age (40 years) and disease duration (DD) (10 years). Expanded Disability Status Scale (EDSS) scores were higher in the NTZ group than in the DMF group at 3.5 vs. 2.5, respectively (*p* = 0.001). However, in both groups, age values correlated with DD (r = 0.42; *p* < 0.001), EDSS (r = 0.52; *p* < 0.001) and age at onset (r = 0.18; *p* < 0.001). Furthermore, age-adjusted Kaplan–Meier curves showed that NTZ-treated subjects maintained a 1.0–3.0 EDSS status score (*p* = 0.003) more frequently and a 3.5–7.0 score (*p* = 0.022) significantly less frequently compared with DMF-treated subjects. The EDSS percentage mean difference between NTZ and DMF groups was 81.6%, decreasing inversely with age (r = −0.34; *p* < 0.001). Finally, high EDSS score values were reached at the age of 39–40 years, regardless of their experimental group. We demonstrated age as a major contributor in disability and response to therapy in current management of MS. Thus, age should be considered in the risk/benefit evaluation in decision making for the disease modifying treatments in MS.

## 1. Introduction

In the epoch of great development of biotechnologies, several disease-modifying therapies (DMTs) have been devised for different clinical phenotypes and for their neurological impact in the management of multiple sclerosis (MS). Thus, mild–moderate MS as well as highly active MS can now be targeted using first-line and second-line therapies, respectively. However, although randomized clinical trials (RCTs) are based on widely accepted scientific research methods, including case–control methods, blinded or double-blinded methodologies and logistic, stepwise or multivariate regression analysis, they present a significant confounding bias: the underestimation of age as the main contributor to disability in MS [1,2,3]. This is also the case for dimethyl fumarate (DMF) and natalizumab (NTZ). Specifically, the age of each patient may be a relevant biological factor determining functional changes in the innate and adaptive immune system, in turn influencing MS course and response to therapy [4,5,6,7].

Age implies known prognostic factors, including disease duration (DD) [8] and a number of experienced therapies: the first correlating with the central nervous system’s (CNS) threshold of pathology and disability [2,3], the second with a suboptimal response to DMTs [9]. Finally, the well-known CNS compartmentalization and associated chronic inflammation induce neurodegeneration [10] and a parallel process called terminal differentiation of immune cells [11]. This causes antigen-specific lymphocytes to proliferate less than T cells in MS patients with a short disease duration, a process called immunosenescence that largely correlates with the response of DMTs [12]. Coherently, MS is known to be induced by inflammatory demyelination and axonal loss [13]; the latter process induces end-stage irreversible neurological impairment and the former induces a reversible relapsing neurological dysfunction in the early stages of the disease [14]. Neuroinflammation decreases over time and is driven by peripheral blood mononuclear cells (PBMCs) [14]. In turn, PBMC infiltrates constitute perivascular plaques, but also ectopic subpial lymphatics, thus assuming a significant role in the compartmentalization and chronicization of inflammation [14,15,16,17]. In the late stage of MS, perilesional PBMCs become rare, unlike the increasing ectopic lymphatics, resulting in the lower efficacy of DMTs [15,16,17]. Acting mainly as anti-inflammatory or immunomodulators, DMTs could be less effective in patients with elevated DD and age [18]. However, it has been widely demonstrated that MS presents very different biological and phenotypical spectrums depending not only on which phase of natural history is being considered but also on age [19,20,21], which is known to be the major contributor to disability resulting from MS [1,2,3].

Irrespective of the observations above, the RCTs evaluated MS not as a biological process evolving over time, but as a continuous statistical variable uniformly distributed in the study population, although it was heterogeneous for age, DD and expanded disability status scale (EDSS) score. Despite these considerations, the concept of the age-related efficacy of DMTs is largely missing in the current literature on MS and demyelinating diseases. Furthermore, the implication of age with regard to the risk/benefit balance in clinical practice of MS, if considered, has focused on the risk rather than the benefit.

NTZ is an engineered IgG4 antibody binding to the very-late-activating antigen 4 and alpha-4/beta-7 integrins, resulting in a sequestering effect; DMF is a chemical species of the Krebs cycle which has an immunomodulating effect acting on nuclear factor (erythroid-derived 2)-like 2 (Nrf2) and the hydroxycarboxylic acid receptor 2 (HCAR2), of which it is an agonist. However, even knowing the pharmacodynamics of these molecules, the correlations between clinical and paraclinical variables that rule the efficacy of these DMTs are still unknown in real-world management of MS.

The primary aim of this study was to evaluate, in a real-world setting, the age-related influence on disability among MS patients treated with DMF compared with those treated with NTZ. Secondarily, we aimed to investigate the correlation between age, age at onset of disease, DD and EDSS in MS patients treated with different lines of therapy. Finally, adjusting for age, age at onset and DD in both treatment groups, we wanted to assess the proportion of paraclinical worsening among the patients expressing high EDSS (≥3.5).

## 2. Results

We observed two study populations: one consisting of 173 patients treated with DMF and another consisting of 94 patients treated with NTZ. The average age, age at onset of disease and DD were similar between the NTZ and DMF groups. The observational period was 36.0 months (range 24.0–36.0). Mean age was 40.0 and 39.2 years, mean age at onset was 28.4 and 29.1 years, and mean DD was 13.7 and 11.4 years, respectively, for NTZ and DMF populations, with no significant differences between groups (mean *p* = 0.447). The average EDSS was 3.5 in the NTZ group and 2.5 in the DMF group. This difference was statistically significant (*p* < 0.001). The sex ratio was 1.3:2.2 in the NTZ-treated group and 1.4:2.7 in the DMF-treated group, in favor of women in both, with no significant difference in the proportion between male and female groups (χ^2^ = 0.425). The two study populations expressed similar annual relapse rates (ARRs) during the observation period, specifically, 0.19 for NTZ- and 0.21 for DMF-treated MS patients (*p* = 0.123); however, the first population had a naive patients proportion of 25% compared with 35% in the second one. Table 1 summarizes demographical data with measures of the central tendency and dispersion of the considered variables. Due to the exclusion criteria, we had 26 unconsidered patients (9.8% of the total).

**Figure 1:**
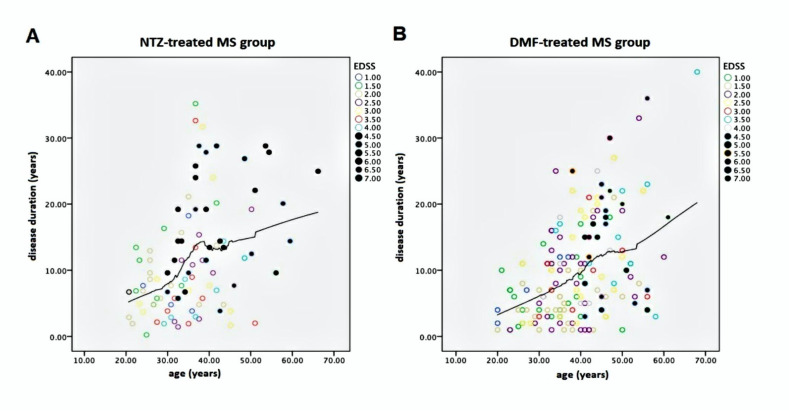
Graphical representations of correlation between age (years) and DD (years) with EDSS-labeled cases from the NTZ-treated (**A**) and DMF-treated (**B**) MS groups. Note, in bold, the EDSS cases ranging from 4.5 to 7.0 with a great slope corresponding to 39 to 40 years. EDSS—Expanded Disability Status Scale; NTZ—Natalizumab; DMF—Dimethyl fumarate; MS —Multiple Sclerosis.

The Spearman rank test evidenced strong statistical correlation between age and DD (r = 0.42; *p* < 0.001), as well as between age and EDSS (r = 0.52; *p* < 0.001). Furthermore, DD correlated with EDSS (r = 0.46; *p* < 0.001), as expected. In all cases, we found a direct and significant statistical correlation between time-dependent variables and EDSS. Consistently, we found a strong direct correlation between the age at onset and age (r = 0.62; *p* < 0.001), DD (r = 0.37; *p* < 0.001) and EDSS (r = 0.18; *p* < 0.001). Figure 1 shows the correlation between age and DD with EDSS-labeled cases from the NTZ- and DMF-treated MS groups.

**Figure 2:**
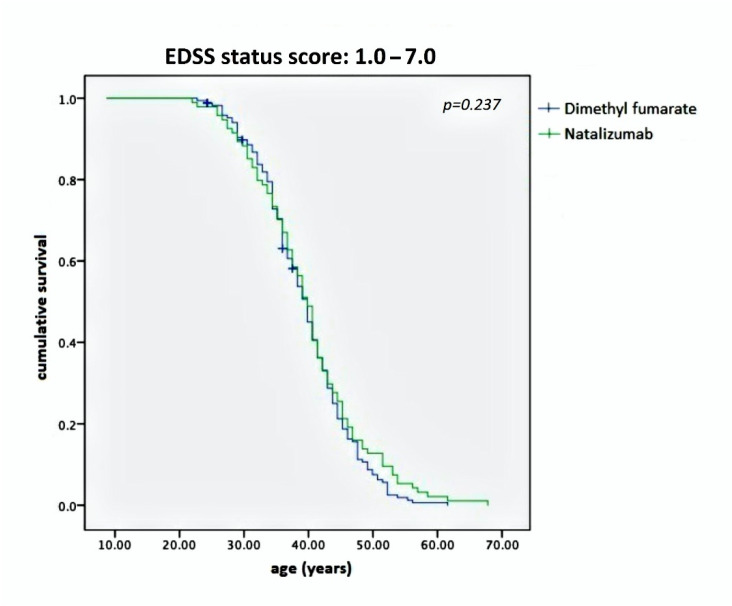
Kaplan–Meier curves comparing reaching an EDSS status score of 1.0–7.0 over the long age period in both NTZ- and DMF-treated MS study populations. Note that the two curves are largely superimposed, indicating the same timing in reaching the functional milestones for NTZ- and DMF-treated patients. That means the two study populations were homogeneous and statistically comparable. EDSS—Expanded Disability Status Scale; NTZ—Natalizumab; DMF—Dimethyl fumarate.

In both graphics of Figure 1, MS patients with high EDSS are localized after an ideal cut-off vertical line, corresponding to ≥39–40 years of age, suggesting an age-dependent threshold effect for reaching EDSS ≥3.5. We utilized Kaplan–Meier curves to compare reaching an EDSS status score of 1–7 over the long age period in both NTZ- and DMF-treated MS study populations. This analysis evidenced no difference between groups but a substantial overlap among them (*p* = 0.237), as shown in Figure 2.

**Figure 3:**
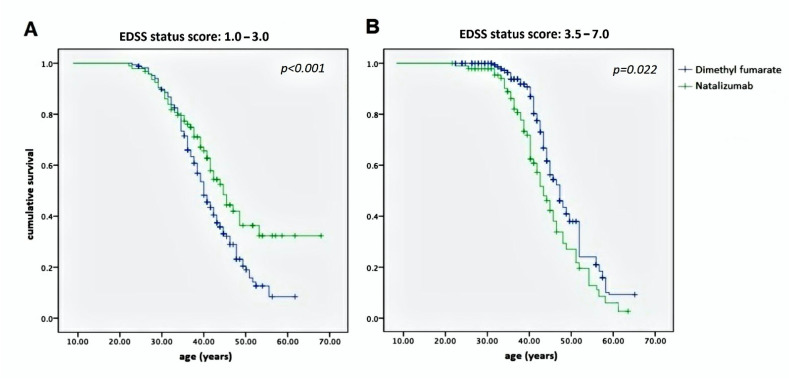
Kaplan–Meier curves representing the proportion of patients with an EDSS status score of 1.0–3.0 (**A**) and 3.5–7.0 (**B**) according to age in both the NTZ- and DMF-treated MS study populations. The highest curve in (**A**) represents the better ability over time of NTZ to maintain the patients at a low grade of disability; the highest curve in (**B**) represents the better ability over time of DMF to stabilize the EDSS in patients that reached a high grade of disability. EDSS—Expanded Disability Status Scale; NTZ—Natalizumab; DMF—Dimethyl fumarate.

However, the same statistical analysis applied to age vs. two ranges of EDSS, 1.0–3.0 and 3.5–7.0, showed evidence of significantly divergent curves, *p* < 0.001 and 0.022, respectively, as shown in Figure 3.

**Figure 4:**
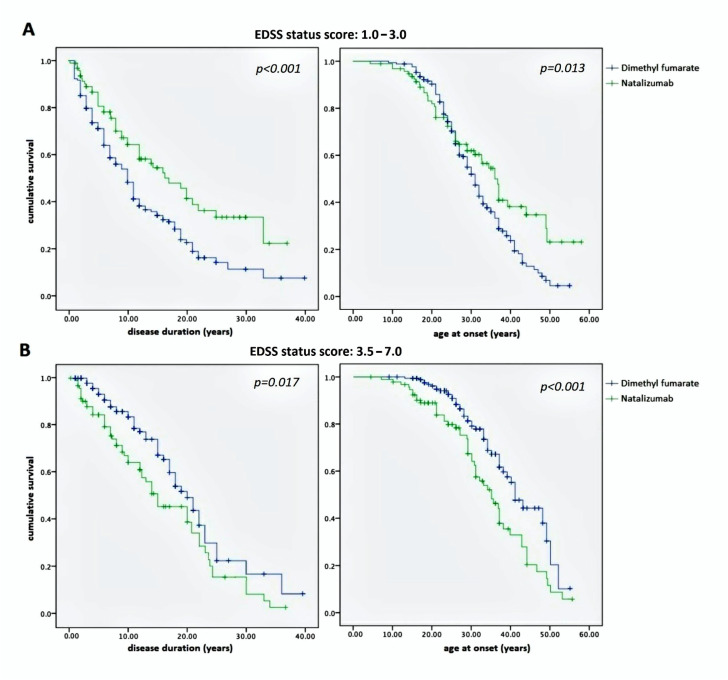
Kaplan–Meier curves representing the proportion of patients with EDSS status scores of 1.0–3.0 (**A**) and 3.5–7.0 (**B**) according to the disease duration (left) and age at onset (right), in both NTZ-treated and DMF-treated MS study populations. (**A**) Note the superiority of NTZ in preventing the disability progression and in maintaining an EDSS of 1.0–3.0 compared with DMF; conversely, (**B**) note the superiority of DMF in maintaining an EDSS of 3.5–7.0 compared with NTZ. EDSS—Expanded Disability Status Scale; NTZ—Natalizumab; DMF— Dimethyl fumarate.

We assessed the same milestones (EDSS score 1.0–3.0 and 3.5–7.0) with respect to the DD and age at onset. In both cases, Kaplan–Meier curves were much more suggestive, confirming strong correlation between both DD and age at onset with disability in reaching EDSS 1.0–3.0 (*p* < 0.001 and *p* = 0.013, respectively) and 3.5–7.0 (*p* = 0.017 and *p* < 0.001, respectively) (Figure 4).

**Figure 5:**
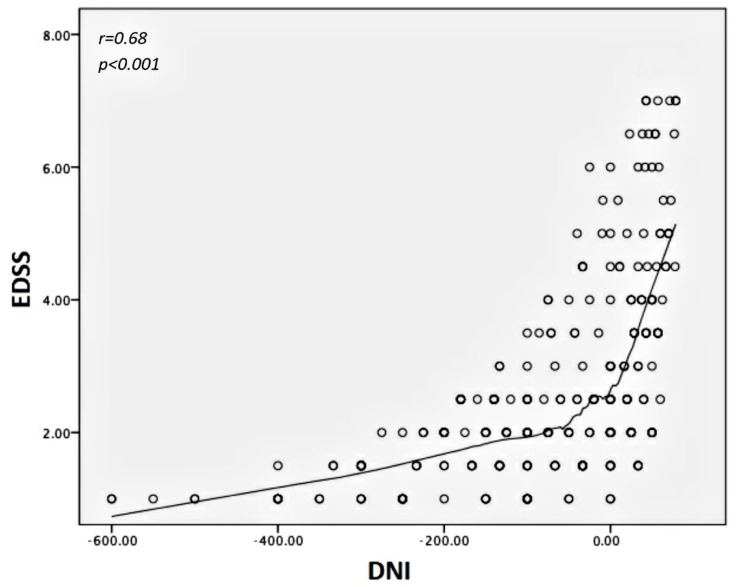
Scatter plot showing statistical correlation between DNI and EDSS scores. Note the increased value of DNI from the low to the high grade of disability, meaning that the main differences in disability between NTZ- and DMF-treated patients are concentrated in the low grade of EDSS. DNI is expressed by the ratio between EDSS scores from patients treated with NTZ and EDSS scores from patients treated with DMF, matched for age, disease duration and age at onset of disease. DNI—Differential Neurological Impairment; EDSS—Expanded Disability Status Scale; NTZ—Natalizumab; DMF—Dimethyl fumarate.

We found the differential neurological impairment (DNI) of the NTZ treatment group increased from—600% to 78.5%, moving from the age of 20 to 55 years, with a corresponding EDSS range of 2.0–7.0 (Figure 5).

**Figure 6:**
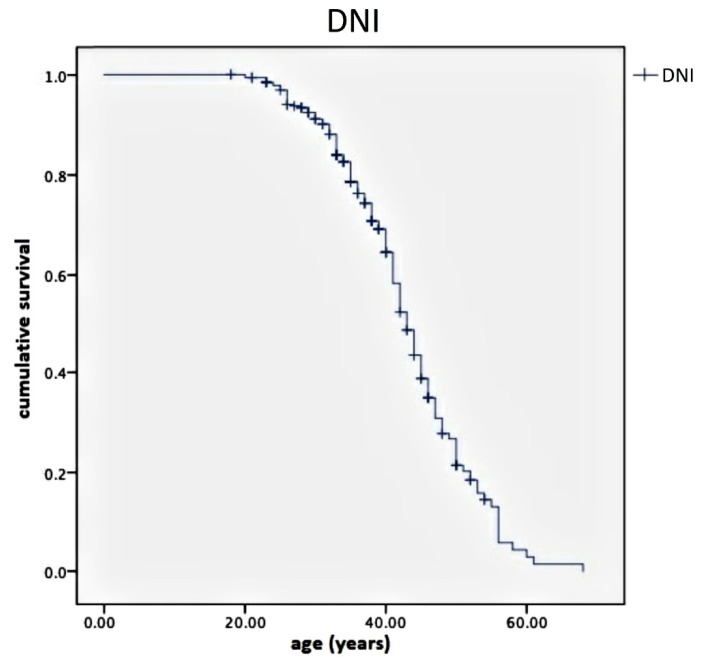
Kaplan–Meier curves showing DNI according to the considered age range. Note the increasing slope curve near the age value of 39 to 40 years. This is the cut-off value indicating the rapid annulment of the disability differences between the patients belonging to the NTZ group and those of the DMF group. DNI is expressed by the ratio between the EDSS of patients treated with NTZ and the EDSS of patients treated with DMF, matched for age, disease duration and age at onset of disease. DNI—Differential Neurological Impairment; EDSS—Expanded Disability Status Scale; NTZ—Natalizumab; DMF—Dimethyl fumarate.

Specifically, the average value of DNI was −81.6%, corresponding to an EDSS value ranging from 1.8 to 2.0, with a 95% CI ranging from−96% to −67.8%. Finally, the Kaplan–Meier curve represents DNI according to the considered age range (Figure 6).

We found that DNI significantly correlated with age, DD and EDSS according to r = 0.34, *p* < 0.001; r = 0.29, *p* < 0.001; and r = 0.68, *p* < 0.001, respectively. Regarding the differential inhibition of activity progression (DIAP), it assumed almost constant values over the entire observational period, corresponding to an average of 51%. The mean age of all MS patients with disease activity was 39.3 ± 8.4 years. A large proportion of these (46%) worsened clinically without increased lesion load; on the other hand, the remaining worsening patients had increased lesion load, with ages ≤39 years and EDSS ≤ 3.5. Specifically, we found about 9% disease activity per year in the NTZ group and 10.6% in the DMF group. Only about 30% of this activity was sustained by the MRI abnormalities in the first case, while more than 60% was sustained in the second.

## 3. Discussion and Conclusions

Age represents a major determinant of disability in MS. However, the on-treatment correlation between these variables in determining the real-life effectiveness of DMT in MS is still not understood.

A similar issue, albeit in the ideal conditions of the RCT, was first investigated by Weideman et al. [18]. To compare the efficacy performances of each DMT in normalized terms, the authors introduced a variable called inhibition of disability progression (IDP) resulting from the percentage ratio between the proportion of patients expressing confirmed disability progression (CDP) in the DMT group and those expressing it in the placebo group. In our study, the absence of the latter hampered the use of IDP; however, we introduced the DNI variable, which expresses the percentage difference between the EDSS of each NTZ-treated patient compared with each DMF-treated patient, and the DIAP variable, which expresses the percentage of progressive NTZ-treated patients compared with that of those treated with DMF.

In a three-year retrospective, cross-sectional, clinical and MRI study, we evaluated the statistical correlation between age, age at onset, DD and EDSS, assessing a real-life population constituted by DMF- and NTZ-treated MS patients. The two study populations had similar and comparable data for all time-dependent variables: DD, age and age at onset; thus, we conducted the statistical analysis assured of a notable adherence to real conditions and without having to consider a study observation period unforeseen by the aims.

We represented both populations with Kaplan–Meier curves concerning DD, age and age at onset. Thus, Figure 2 shows a wide overlap among these curves. The only remarkable difference was found in the NTZ-treated group, which was significantly impaired compared with the DMF-treated group, having an EDSS value of 3.5 vs. 2.5, respectively. This difference is consistent with the so-called second-line DMTs, reserved for patients suffering from a high-activity disease type.

The first finding is crucial for acquiring a general value, demonstrating a strong correlation between age, age at onset, DD and EDSS. These data appear to be reliable, since they were verified in both study populations separately. Stepwise regression analysis also proved the predictability of age with respect to the EDSS. Translated into physiopathological terms, this means that the prescription of DMT in MS induces a different effect according to the patient’s age and DD. Specifically, at a mean age of 39 years, we found more disabled and paraclinically free patients with high EDSS (Figure 1) who were largely prone to disability progression on the neurodegenerative basis. It is known that MS is a memoryless pathology, reaching high EDSS regardless of the disease burden experienced before reaching the EDSS score of 3.0. This was also confirmed by Weinshenker [22] in studies of the natural history of MS. This author indicated an EDSS score of 3.0 or 4.0 as the cut-off value for secondary progressive MS and irreversible disability progression. Therefore, the prescription of DMT in MS patients expressing elevated DD or age is likely to have suboptimal efficacy in terms of inhibition of disease progression. This statement is verified by both our study populations, as Figure 1 shows the EDSS scores of 4 behind the ideal vertical line corresponding to 39 to 40 years of age. Like age, the age at onset and the DD express good correlation with respect to EDSS in both populations. Coherently, elevated DD statistically predicted high disability, despite DMT. In our study, an age ≥39 years corresponded to an average DD of 10 years. In effect, in both populations and among patients that exceeded these cut-off values, we observed a proportion of 46% disability progression not associated with clinical or paraclinical relapses.

The Kaplan–Meier curves showed no difference between the two study populations with regard to the overall disability evolution from EDSS 1.0 to 7.0, as represented in Figure 2. However, significant differences concerning the achievement of EDSS milestones were noted. In effect, Figure 3A shows a net statistical advantage in maintaining EDSS 1.0–3.0 in NTZ-treated MS patients compared with those treated with DMF; in addition, a larger proportion of patients achieving an EDSS of 3.5–7.0 belonged to the DMF-treated group, as shown in Figure 3B.

Although the interpretation of curves in Figure 2 should consider the regression toward means, we think that they indicate a substantial equivalence between NTZ and DMF in preventing disability progression during the lifetime period. This does not mean that the two DMTs had the same efficacy, but they did have the same stark effect on the two different study populations: one characterized by mild–moderate MS treated with a first-line DMT, namely DMF, and the other by high-activity MS treated with a second-line DMT, namely NTZ. Taking this into account, a remarkable difference was identified between them: NTZ demonstrated a better ability to maintain a low-grade EDSS score than DMF (Figure 3A) but not to prevent a high one (Figure 3B). The latter produced a higher curve than NTZ, meaning that the DMF-treated patients were more persistently in the EDSS 3.5–7.0 range. Similar observations were also obtained by assessing disability progression with respect to the DD and age at onset (Figure 4). In both cases, NTZ was more effective in maintaining a low EDSS score (Figure 4A), while DMF showed superiority in preventing disability progression at high EDSS scores (Figure 4B). These observations agree in suggesting a neuroprotective effect of DMF, which is more evident in the late stage of disease when degenerative damage occurs; this is unlike NTZ, which demonstrates its main efficacy in the early stage, when the neuroinflammation is prominent [23]. This interpretation is in accordance with the two-stage evidence of disability progression in MS, enriching our knowledge about the mechanisms of action of NTZ, as a DMT that is particularly effective in the early phase of disease, and of DMF in the late phase.

The efficacy of NTZ in disability prevention is also confirmed by its DIAP, which evidenced a three-year disease activity reduction of 51% in NTZ-treated patients. From analyzing DNI, we know that the main difference in EDSS values between patients undergoing the second-line DMT compared with those receiving first-line DMT was inversely related to age (Figure 5) and DD, but it was also a measurement that rapidly decreased with disability, resulting in an inverse correlation to the same EDSS score (Figure 6). The DNI curve represents the difference in terms of ratio between the disability of NTZ-treated patients compared with those treated with DMF. Therefore, the left side of the curve corresponds to higher values of difference (near to one), where age values are far from 39–40 years; the descending right side of the curve assumes low values (tending to the zero), where age values are near or above 40 years. This means the differences in neurological disability between the first- and second-line DMTs are detectable only before a determined age threshold but not after. We found this age threshold to be about 40 years old.

In effect, the average age of worsening patients was 39.3 years and that of clinically progressive patients (without MRI activity) was >39 years. The main proportion of these patients experienced disability progression without the classical relapse, especially in the NTZ group.

We recognize as a limitation of this study the lack of data stratification by gender. This topic represents an important field in the current literature, so it would require a separate discussion. However, we can state from preliminary results that the correlation analysis between disability and time-dependent variables was unaffected by gender (data not shown).

Our findings, although expected based on clinical experience, have not been formally demonstrated in a real-life setting until now but are consistent with the conclusions from the recent work of Ann Marie Weideman et al. [18]. These findings, in both ideal conditions and our real-life setting, demonstrated that the prescription of a DMT in MS must be as early as possible to achieve the maximum effectiveness from first-line or second-line treatment. Thus, it must consider the age, age at onset of disease and DD of the patient in the treatment choice. Age and DD represent not only major determinants in disability, but also in the response to therapy in MS. Specifically, 39.3 years of age represents a cut-off value beyond which patients’ disabilities are prone to evolve, regardless of the DMT. In conclusion, our data strongly support considering age in the evaluation of risk/benefit for decision making concerning DMT in MS.

## 4. Materials and Methods

*Study population*: we enrolled all MS patients treated with DMF and NTZ afferent at the Multiple Sclerosis Centre of the Neurological Department at the “F. Ferrari” Hospital in Casarano, Lecce (Italy), the “A. Perrino” Hospital in Brindisi (Italy), and the “V. Fazzi” Hospital in Lecce (Italy). These MS centers are located in the Salento peninsula in the extreme south of Italy and the “F. Ferrari” Casarano Hospital was used as a headquarters for data collection and coordination of clinical activities in the present study.

All patients were enrolled according to the following inclusion criteria: signature on written informed consent; diagnosis of clinically defined MS (McDonald criteria, revised in 2017 by Polman) without limitation of DD, age ranging from 20 to 70 years and EDSS scores ranging from 1.0 to 7.0; baseline MRI of the brain and spinal cord performed no more than three months before study entry; regular follow-up with quarterly neurological assessment started at least three months before the study entry.

The exclusion criteria were all metabolic and complicated internal comorbidities, including dysthyroidism, diabetes mellitus, lung and heart dysfunctions resulting in obstructive/restrictive syndromes and heart failure syndromes, as well as all neuromuscular confounding comorbidities, including myasthenia gravis, skeletal deformities, etc. These chronic comorbidities are known to affect all domains of physical functions as well as quality of life, so we excluded them from the study to not affect the neurological disability evaluation. We admitted all other comorbidities not associated with organ damage, for example, uncomplicated dysthyroidism or diabetes, atrial fibrillation without heart failure, etc. In addition, we excluded the drop-out subjects. These patients discontinued DMT treatment early due to adverse reactions, poor tolerance to the drug, or not reaching the minimum follow-up period of 24 months needed for the enrollment. We did not consider the patients expressing EDSS ≥7.5 since these subjects frequently suspend therapy and present difficulties in accessing the hospital. Coherently, each enrolled patient exceeding the EDSS score of 7.0 was considered statistically lost to the follow-up.

*Study design*: this was an open-label, clinical and paraclinical multicenter, retrospective and cross-sectional study that started in May 2017 and ended in May 2020. All MS patients enrolled had completed at least three months of treatment with DMF or NTZ, and underwent the neurological assessment with quarterly EDSS calculation as well as brain and spinal cord MRIs every year. To make the two study populations homogeneous for investigation purposes, we divided them according to increasing age ranks of five years each, ranging from 20 to 70 years. All EDSS scores and subscores increasing by 0.5 points, detected at two consecutive neurological assessments, were considered as significant indicators for CDP. Equally, all increasing lesion load resulting from new T2-weighted or T2-weighted enlarging lesions and the appearance of contrast enhancing lesions in the MRIs were considered indicators for paraclinical activity of worsening.

*Considered variables*: the considered variables included age, age at onset of disease, DD and EDSS. Moreover, the proportion of paraclinical worsening among progressive patients expressing an EDSS score was ≥3.5. Unlike the routine clinical management, the EDSS assessment and MRI evaluations were performed by the same experienced operator (RDM) over the entire study duration. We preferred this approach to the double assessment one as it made it possible to simplify the data interpretation, avoiding the confounding factor concerning the agreement of statistics between the first and second operator.

*Statistics*: we used version 24.0 of the Statistical Package for Social Science (SPSS 24.0) for descriptive and nonparametric inferential statistics. Specifically, we used the Mann–Whitney U-test (*p* < 0.05) to calculate the differences between means of considered variables and the Spearman rank test to obtain the correlation between them. Furthermore, age-adjusted Kaplan–Meier curves were used to represent the proportion of patients in the 1.0–3.0 and 3.5–7.0 EDSS ranges over different age intervals. Finally, we considered DNI as a variable indicating the percent difference in EDSS between each of the NTZ(EDSS^ntz^)- and DMF (EDSS^dmf^) -treated MS patients with the same mean age at onset, DD and age rank ranges. DNI was defined according to the following equation:

$$\mathrm{DNI}=\left(1-\frac{{\mathrm{EDSS}}^{\mathrm{ntz}}}{{\mathrm{EDSS}}^{\mathrm{dmf}}}\right)\times 100$$

This variable ranges from values inferior to zero, i.e., from negative to zero, to positive fractions of one, according to the denominator amount. Therefore, DNI indicates the percentage difference in EDSS of each NTZ-treated MS patient compared with each DMF-treated MS patient, both having similar age and DD rank ranges. A negative DNI value indicates an NTZ-treated patient who is more disabled compared to a DMF-treated one; DNI = 0 means no difference; DNI equal to a positive fraction indicates a DMF-treated MS patient who is more disabled compared to an NTZ-treated one. The distribution of this variable in the study population was also important for studying age and DD as contributors to disability in MS patients with respect to whether they were treated with first- or second-line DMT. We applied DNI to matched subjects, so that they belonged to the same rank of age, DD, and age at onset, to obtain an internal data normalization. In this way, we made the EDSS from each NTZ-treated MS patient statistically comparable with the EDSS from a corresponding DMF-treated patient. Coherently, the difference in neurological impairment between one patient treated with NTZ and another treated with DMF became a numeric and continuous variable of the DNI, enabling us to assess the distribution among the study population according to time-dependent parameters (age, DD, etc.). Finally, the two compared populations, although numerically unbalanced, were statistically comparable as they were divided into ranks, where each rank comprised patients having similar values of time-dependent variables and, accordingly, DNI. Although NTZ-treated patients were about half in number compared to DMF-treated ones, they constituted two comparable populations since the time-dependent variables were distributed equally among the two groups.

One variant of the DNI formula was applied to the proportion of patients that manifested clinical or MRI activity during the three-year observational period in the NTZ group compared with those in the DMF group to indicate the DIAP. DIAP was defined according to:

$$\mathrm{DIAP}=\left(1-\frac{A^{\mathrm{ntz}}}{A^{\mathrm{dmf}}}\right)\times 100$$

where A^ntz^ and A^dmf^ indicate the annual proportion of patients that experienced CDP or MRI activity in the NTZ and DMF groups, respectively. DIAP expresses, as a percentage, the differential ability of NTZ to prevent disease activity or clinical progression compared with DMF in each observational year of the study. Therefore, DIAP is a clinical–paraclinical composite variable.

## Figures and Tables

**Table 1:** Demographic and pathological characteristics of treated MS populations. DD—Disease Duration; EDSS—Expanded Disability Status Scale; ARR—Annual Relapse Rate; MS—Multiple Sclerosis.

	Natalizumab Mean ± SD (95% CI)	Dimethyl Fumarate Mean ± SD (95% CI)	*p*
**Age** (years)	40.0 ± 11.1 (37.8–42.5)	39.2 ± 9.3 (38.0–40.0)	0.482
**Age at onset** (years)	28.4 ± 11.2 (26.1–30.7)	29.1 ± 8.9 (28.1–30.0)	0.413
**DD** (years)	13.7 ± 11.8 (10.0–13.6)	11.4 ± 7.6 (9.1–10.7)	0.095
**EDSS**	3.5 ± 1.6 (3.2–3.9)	2.5 ± 1.7 (2.3–2.6)	<0.001
**ARR**	0.19 ± 0.02 (0.16–0.21)	0.21 ± 0.01 (0.19–0.23)	0.123

## Data Availability

The data presented in this study are available within the article.

## Abbreviations

CDP	confirmed disability progression
DD	disease duration
DIAP	differential inhibition of activity progression
DMF	dimethyl fumarate
DMTs	disease-modifying therapies
DNI	differential neurological impairment
EDSS	expanded disability status scale
MS	multiple sclerosis
NTZ	natalizumab
PBMCs	peripheral blood mononuclear cells
RCT	randomized clinical trials
